# Impedance of Extracellular Fluid, Volume, and Local Tissue Water Can Be Reliably Measured in People With Lower Limb Lymphedema

**DOI:** 10.1093/ptj/pzac025

**Published:** 2022-02-28

**Authors:** Charlotta Jönsson, Karin Johansson, Maria Bjurberg, Christina Brogårdh

**Affiliations:** Department of Health Sciences, Lund University, Lund, Sweden; Department of Hematology, Oncology and Radiation Physics, Skåne University Hospital, Lund, Sweden; Department of Health Sciences, Lund University, Lund, Sweden; Department of Hematology, Oncology and Radiation Physics, Skåne University Hospital, Lund, Sweden; Department of Clinical Sciences, Lund University, Lund, Sweden; Department of Health Sciences, Lund University, Lund, Sweden; Department of Neurology, Rehabilitation Medicine, Memory Disorders and Geriatrics, Skåne University Hospital, Lund, Sweden

**Keywords:** Intrarater Reliability, Lower Limb, Lymphedema, Outcome Measures

## Abstract

**Objective:**

Lower limb lymphedema (LLL) is a chronic condition. To be able to evaluate changes of LLL over time and effects of interventions, reliable measurement methods are important. Currently, there is limited knowledge of the reliability of commonly used measurement methods in LLL. The study objective was to evaluate the test–retest (intrarater) reliability of impedance of extracellular fluid, volume, and local tissue water measurements in people with unilateral or bilateral LLL and measurement errors both for a group of people and for a single individual.

**Methods:**

Forty-two people with mild to moderate unilateral or bilateral, primary or secondary LLL were measured twice, 2 weeks apart. Impedance of extracellular fluid was measured by bioimpedance spectroscopy and calculated as arm-to-leg ratio, volume with circumference measurements every 4 cm, and local tissue water with tissue dielectric constant at 14 points. Test–retest reliability was evaluated using the intraclass correlation coefficient [ICC(2,1)], changes in the mean, SE of measurement in relative terms (SEM%), and the smallest real difference in relative terms (SRD%).

**Results:**

For the impedance ratio, the reliability was high [ICC(2,1) = 0.79–0.90] and the measurement errors were acceptable (SEM% = 5.0%–5.2%; SRD% = 14.0%–14.4%). For volume, the reliability was high (ICC = 0.99) and the measurement errors were low (SEM% = 1.1%–1.7%; SRD% = 3.1%–4.6%). For the tissue dielectric constant, the reliability was fair to excellent [ICC(2,1) = 0.68–0.96] and the measurement errors were acceptable (SEM% = 4.2%–9.7%; SRD% = 11.7%–26.8%).

**Conclusions:**

Measurements of impedance of extracellular fluid, volume, and local tissue water are reliable in people with mild to moderate LLL. The measurement errors were acceptable in all 3 methods indicating that real, clinical changes in lymphedema can be measured both for a group of people and a single individual.

**Impact:**

The results from this test–retest reliability study can help clinicians and researchers to interpret if real clinical changes in lymphedema occur over time or after an intervention in people with mild to moderate LLL.

## Introduction

Lymphedema is considered a chronic condition and is the result of the accumulation of interstitial fluid due to impairment of the lymphatic system. Lymphedema is divided into primary lymphedema, caused by congenital malformation of the lymphatic system, and secondary lymphedema, caused by cancer treatment, trauma, or repeated infections affecting the lymphatic system.^1^

Assessments of limb volume is very common in lymphedema management.^1^^,^^2^ The total leg volume can be measured using the water displacement method,^2^ the optoelectronic measurement method,^3^ or the tape measurement method.^4^ Of these methods, the tape measurement method is the most commonly used^2^^,^^5^^,^^6^ due to its simplicity and low cost. However, assessments of swelling in lower limb lymphedema (LLL) can be problematic, because there can be bilateral involvement in both primary LLL and secondary cancer-related LLL. To address this issue some studies have suggested that each limb should be evaluated separately.^2^^,^^5–7^ Together with volume measurements, other clinical assessments, such as palpation of skinfold thickness^8–10^ and recordings of a patient’s subjective experience of heaviness and tightness in the limbs with lymphedema,^11–13^ are used to provide broader clinical information of treatment-related changes in lymphedema.

In lymphedema management repetition of measurements over time is common. It is therefore of great importance to determine if a change in lymphedema measurements is due to a treatment effect or to an inherent variation. For a method to be useful it needs to have a high test–retest reliability with small or acceptable measurement errors.^14^

Overall, few studies have evaluated the test–retest reliability of volume measurements based on circumference measurements every 4 cm along the lower limb.^6^^,^^7^^,^^15^ Existing studies have shown small intrarater variability,^6^ excellent internal consistency compared with perometry,^15^ or high agreement with small measurement errors.^7^ However, in these studies, people who were healthy were included. Hence, there is a lack of knowledge regarding the reliability of volume measurements based on circumference measurements in people with LLL.

Bioelectrical impedance spectroscopy (BIS) is a method to assess the presence of excess lymph in the affected limb relative to that of the unaffected limb.^16^^,^^17^ BIS assesses the electrical resistance (impedance) through the body at different frequencies.^18^ Based on normal impedance values of people who are healthy, thresholds for lymphedema of the lower limbs have been calculated.^19^ Recently, the use of the arm-to-leg impedance ratio has been suggested as a suitable method for identifying bilateral lymphedema.^20^ However, until now it was unknown whether this impedance ratio can be reliably measured in people with LLL.

The tissue dielectric constant (TDC) method uses high-frequency electromagnetic waves to measure local tissue water in the skin. An advantage of this method is that each predefined point can be evaluated separately, making the method more useful when a bilateral involvement of lymphedema is present. Yet, there is only 1 study of people who were healthy that has evaluated the reliability of many predefined points on the thigh and calf.^7^ Thus, there is a lack of knowledge about the reliability of TDC measurements in people with LLL. Therefore, the aim of this study was to evaluate the test–retest (intrarater) reliability of impedance of extracellular fluid (ECF), volume, and local tissue water measurements in people with unilateral or bilateral LLL and measurement errors both for a group of people and for a single individual.

## Methods

### Research Design

A test–retest intrarater reliability design was used in the present study, and the COnsensus-based Standards for the selection of healthy Measurement INstruments (COSMIN) checklist was used for guidance.

### Participants

Forty-two people with LLL were recruited from the lymphedema unit at Skåne University Hospital from April 2018 to March 2019. The 5 inclusion criteria were as follows: age 18 years or older; a diagnosis of unilateral or bilateral primary or secondary LLL; persistent lymphedema for the last 6 months; a stable volume of the lower limbs for the last 6 months (ie, a total limb volume variation <5% for each limb); and treatment with compression stockings during the day or during the day and the night according to usual care. To further ensure a stable limb volume over time the compression garment had not to be older than 2 months when included in the project.

The 6 exclusion criteria were as follows: ongoing treatment to reduce the limb volume; circulatory disorders, such as heart failure, kidney disease, and postthrombotic swelling; prosthetic knee or hip implants; muscular disorders of the lower limbs; intake of diuretic drug or any other drug interfering with the volume of the lower limbs; and inability to understand written or oral information. The diagnosis of primary LLL was based on lymphoscintigraphy, and the diagnosis of secondary LLL was set by a medical specialist before referral to the lymphedema clinic.

Before inclusion all participants received written and oral information about the study and gave written consent to participate.

### Measurements

#### Clinical Characteristics

Body mass index was calculated (kg/m^2^) using the weight measured on a digital scale with an accuracy of ±0.1 kg and the body height reported by each participant.

Thickness of the subcutaneous tissue^21^ of the lower limbs was assessed with the participant in the supine position with bent knees. The palpation was performed by pinching the subcutaneous tissue^8^ using the thumb and index finger at the following sites: dorsal, lateral, and medial side of the lower part of the limbs; and lateral, ventral, and medial side of the upper part of the limbs. Presence of increased thickness was noted as yes or no.

Experience of heaviness and tightness in the limb or limbs affected by lymphedema over the past week was rated using a 100-mm visual analog scale^22^ ranging from “no discomfort” (0 mm) to “worst imaginable discomfort” (100 mm).^11–13^

Leisure time physical activity status during the last 6 months was rated using a 6-graded classification system for physical exercise^23^ that covers the range from a very low level of physical activity to regular very strenuous activity. This classification system has been validated for a Scandinavian population.^23^

#### Measurement Methods for Test–Retest (Intrarater) Reliability

Impedance of ECF was assessed by BIS using a SEAC SFB7 monitor (Impedimed, Brisbane, Queensland, Australia) and the arm-to-leg ratio was calculated. The BIS technique uses a tetrapolar electrode arrangement with 2 measurement electrodes positioned one at each end of the segment to be measured, and 2 drive electrodes each positioned distal to the measurement electrodes. The low-level current is passed between the 2 drive electrodes and the measurement electrodes record the segment’s impedance (*R*).^18^ The resistance, corresponding to ECF (*R*_0_) and to total body fluid (*R*_inf_), was determined, and intracellular fluid (*R*_i_) was calculated.^18^ Arm-to-leg impedance ratio was calculated for each person, using the formula: dominant arm *R*_0_/dominant leg *R*_0_ and nondominant arm *R*_0_/nondominant leg *R*_0_, respectively.^20^ Side of dominance was defined by the dominant arm.

Lower limb volume using circumference measurements every 4 cm was calculated with the truncated cone method.^4^ The measurement method is described in detail by Jönsson et al^7^; in that study, reliability was shown to be high [ICC(2,1) = 0.99] and measurement error was shown to be small in women and men who were healthy.

Local tissue water was assessed by TDC using a MoisturemeterD with an M25 probe having an effective depth of 2.5 mm (Delfin Technologies Ltd, Kuopio, Finland). The measurement method is described in detail by Jönsson et al.^7^ Fourteen measuring points were chosen, intended to cover many different parts of the limb.^7^ Hair was removed with a shaver if necessary, according to the manufacturer’s recommendations. The reliability of these 14 measuring points was shown^7^ to be fair to excellent [ICC(2,1) = 0.63–0.93] in women who were healthy, and poor to excellent [ICC(2,1) = 0.21–0.89] in men who were healthy. The measurement error was acceptable for all points in women and for almost all points in men.^7^

#### Procedure

Each participant was measured on 2 occasions, 2 weeks apart, by an experienced physical therapist (C.J.). The measurements were performed during the morning at about the same time and with the same procedure. Prior to each test occasion the participants were asked to maintain a similar activity schedule in the morning and to empty the bladder.

At each test occasion, shoes, trousers, socks, and compression stockings were removed, and the body weight was measured. The body height was recorded at the first test occasion. Then the participants rested for 10 minutes in a supine position with the legs apart. During the rest, participant characteristics were collected. Measurements of impedance of ECF, volume, and local tissue water were conducted in the same order on each test occasion: first on the right limb and then on the left limb. For local tissue water measurements on the dorsal side of the limbs, the participant turned over to the prone lying position.

#### Measurements of Impedance of ECF

To assess the impedance of ECF, the electrode positions followed the recommendations for the upper limbs, that is, on the dorsal side of the wrists at the level of the process of the radial and ulnar bones,^18^ and for the lower limbs on the dorsal side of the foot midway between the malleoli.^19^ The drive electrode sites were 5 cm distal to the above-described positions, namely, on the dorsal side of the third metacarpal bone and the third metatarsal bone, respectively.^18^ The skin at the electrode sites was cleaned with an alcohol wipe before the application of the gel electrodes. Each limb segment was measured once on each test occasion, and the resistances corresponding to ECF (*R*_0_) were noted from the device display.

#### Measurements of Volume

To assess volume, measuring points for circumference measurements every fourth centimeter were identified and marked using a 110-cm measuring board, a 20-cm ruler, measuring tape, and a water-soluble pen. The foot and heel were placed against the footplate, and markings were made on the lateral side of the limb with the short end of the ruler on the measuring board at each distance,^7^ starting 10 cm above the heel and ending near the groin. Circumference measurements to the nearest millimeter were taken once at each marking by placing the measuring tape close to the skin.

**Figure 1:**
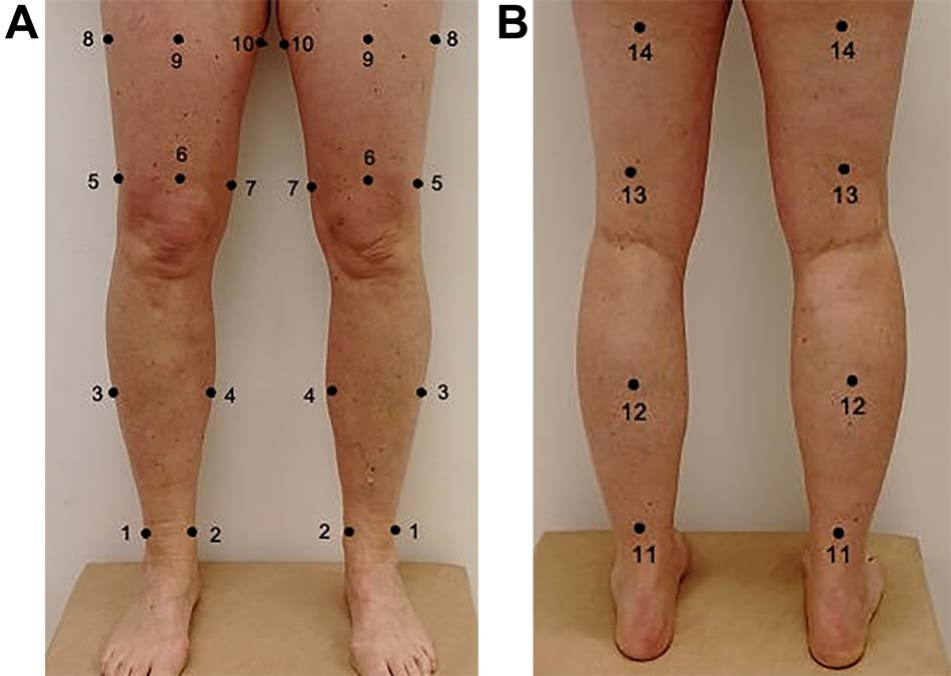
Ten points for tissue dielectric constant (TDC) measurements on the lateral, ventral, and medial sides of the lower limbs (A) and 4 points on the dorsal side of the lower limbs (B).

**Table 1:** Locations of Measuring Points for TDC^a^

**Point(s) (P)**	**Location**
P1 and P2	Calf: 15 cm proximal to the heel, on the lateral and medial sides
P3 and P4	Calf: 30, 35, or 40 cm proximal to the heel, on the lateral and medial sides
P6	3 cm proximal to the base of the patella
P5 and P7	Lateral and medial sides of P6
P9	On a straight line between P6 and ASIS at 15, 20, or 25 cm proximal to P6
P8 and P10	Lateral and medial sides of P9
P11	On the dorsal calf between P1 and P2
P12	On the dorsal calf between P3 and P4
P13	On the dorsal thigh between P5 and P7
P14	On the dorsal thigh between P8 and P10

^a^ ASIS = anterior superior iliac spine; TDC = tissue dielectric constant.

#### Measurements of Local Tissue Water

To assess local tissue water 14 points for TDC measurements were identified and marked using a measuring board, a ruler, a tape measure, and a pen. Markings were made on the lateral, ventral, medial ,and dorsal side of each limb (Fig. 1A,B). The points are shown in Table 1 and described in more detail in the article by Jönsson et al.^7^

TDC measurements were taken in triplicate at each point,^24^ and the average of the 2 closest values was used in the analysis. The identified points for each participant were used on the second test occasion.

### Data Analysis

For statistical analysis, IBM SPSS Statistics version 24 (IBM, Armonk, New York, USA) was used. Demographics and clinical characteristics of the participants are presented as frequencies, means, and SDs or as medians, minimums, and maximums. Measurements of impedance ratio, volume, and local tissue water are presented as means and SDs.

The test–retest reliability analyses comprised agreement between the measurements, systematic changes in the mean, and measurement errors.^14^ Agreement between the measurements was analyzed with ICC(2,1) values. According to Fleiss,^25^ ICCs below 0.40 represent poor reliability, values between 0.40 and 0.75 represent fair to good reliability, and values above 0.75 represent excellent reliability.

Changes in the mean were analyzed by calculating the mean difference between the 2 test occasions (test occasion 2 minus test occasion 1) and the 95% CI for the mean difference ($d$). The 95% CI for the mean difference was calculated to detect any systematic differences between the values from the 2 test occasions. No systematic change in the mean is present if 0 is included in the 95% CI.^26^

The standard error of measurement (SEM) and the smallest real difference (SRD) were used to assess measurement errors. The SEM gives the limit for the smallest change that indicates a real change for a group of people^27^ and is defined as follows^14^: SEM = $\mathrm{SD}{(1-\mathrm{ICC})}^{0.5}$. The SRD represents the limit for the smallest change that indicates a real change for a single person and is defined as follows^27^: SRD = 1.96 × SEM × $\sqrt{2}$. To make the results easier to interpret, the relative terms (SEM% and SRD%, respectively) were also calculated, as follows^27^: SEM% = (SEM/mean) × 100; and SRD% = (SRD/mean) × 100.^28^ An acceptable measurement variability for a group of people (SEM%) is considered to be less than 10%, and that for a single individual (SRD%) is considered to be less than 30%.^28^

Initially, reliability was calculated separately for the women (n = 30) and for the men (n = 12). Because no discernible systematic differences between the sexes were found in the analyses, data for the participants were combined.

Bland-Altman graphs were also plotted to visually demonstrate any systematic bias or outliers. Differences between measurements from the 2 test occasions (test occasion 2 minus test occasion 1) were plotted against the mean of the 2 test occasions for each participant,^26^^,^^27^ together with the 95% limits of agreement.

### Role of the Funding Source

The funders played no role in the design, conduct, or reporting of this study.

## Results

### Participants

The characteristics and demographics of the 42 participants (30 women and 12 men) are shown in Table 2. Thirty of them had secondary lymphedema, mainly due to treatment for gynecological cancer (n = 17). Unilateral involvement (n = 24) was most common, and the duration of the lymphedema varied from 1 year to 40 years. More participants experienced a feeling of heaviness (n = 18) than of tightness (n = 8) in the more affected limb. Palpated thickness in the more affected limb was common both in the lower part of the limb (n = 35) and in the upper part of the limb (n = 33). An impedance ratio exceeding the cutoff values for the diagnosis of lymphedema^20^ was present in 38% (n = 16) of the participants. A volume difference of more than 5% was found in 67% (n = 16) of the participants with unilateral lymphedema. A TDC measurement exceeding the mean + 3 SDs^7^ was present in 74% (n = 31) of the participants in at least 1 point of the more affected limb. The physical activity status varied widely (Tab. 2).

**Table 2:** Characteristics of 42 Participants With LLL^a^

**Characteristic**	**Value**
Sex, women/men, n (%)	30 (71)/12 (29)
Age, y, mean (SD)	61 (14)
BMI, kg/m^2^, mean (SD)	27 (5)
Primary/secondary lymphedema, n (%)	12 (29)/30 (71)
Duration of lymphedema, mo, mean (SD)	130 (92)
Diagnosis, n (%)	
Gynecological cancer/melanoma/urological cancer/other	17 (40)/5 (12)/4 (10)/4 (10)
Lymphedema, bilateral/unilateral, n (%)	18 (43)/24 (57)
By BIS, arm-to-leg ratio, n (%)*^b^*	
MA limb/LA limb	16 (38)/4 (10)
By TDC, n (%)*^c^*	
MA limb, in 1 point/in 2 points or more	6 (14)/25 (60)
LA limb, in 1 point/in 2 points or more	8 (19)/8 (19)
Unilateral lymphedema (n = 24)	
By BIS, interleg ratio, n (%)*^d^*	9 (38)
Volume difference, n (%)*^e^* <5%/≥5% to <10%/≥10% to <20%/≥20% to <30%	8 (33)/6 (25)/7 (29)/3 (13)
Heaviness, n (%)/median (minimum, maximum)*^f^*	
MA limb	18 (43)/35 (13, 75)
LA limb	3 (7)/40 (18, 53)
Tightness, n (%)/median (minimum, maximum)*^f^*	
MA limb	8 (19)/43 (17, 67)
LA limb	1 (2)/67
Location palpated thickness, n (%)	
Lower leg, MA limb	35 (83)
Lateral/dorsal/medial	21 (50)/31 (74)/29 (69)
Lower leg, LA limb	3 (7)
Lateral/dorsal/medial	1 (2)/1 (2)/3 (7)
Upper leg, MA limb	33 (79)
Lateral/ventral/medial	21 (50)/24 (57)/27 (64)
Upper leg, LA limb	3 (7)
Lateral/ventral/medial	2 (5)/0/1 (2)
Graded classification system for physical exercise (scores: 1–6), median (minimum, maximum)*^g^*	4 (2, 6)
Working/retired, n (%)	22 (52)/20 (48)
Sedentary or active job: most sedentary/both sedentary and active/most active, n (%)*^h^*	12 (29)/2 (5)/8 (19)

^a^ BIS = bioimpedance spectroscopy; BMI = body mass index; LA = less affected; LLL = lower limb lymphedema; MA = more affected; TDC = tissue dielectric constant.

^b^ BIS ratios exceeding cutoffs for the diagnosis of lower limb lymphedema.^20^

^c^ TDC values exceeding mean + 3 SDs in people who were healthy.^7^

^d^ BIS ratios exceeding cutoffs for the diagnosis of unilateral lower limb lymphedema.^19^

^e^ {[(Volume of the affected limb minus volume of the unaffected limb)/volume of the unaffected limb] × 100}.^2^

^f^ The experience of heaviness and tightness during the last week, using a visual analog scale.^22^

^g^ The Frändin-Grimby Activity Scale.^23^

^h^ Question about job activity.

**Table 3:** Measurements of Impedance Ratio, Volume, and TDC in 42 Participants With LLL on 2 Test Occasions^a^

**Measurement**	**Mean (SD) on Test Occasion:**	
	**1**	**2**
Impedance ratio		
MA limb*^b^*	1.287 (0.214)	1.321 (0.241)
LA limb*^b^*	1.160 (0.128)	1.178 (0.172)
Volume, mL		
MA limb	9371 (1549)	9372 (1553)
LA limb	8685 (1533)	8705 (1567)
TDC P1		
MA limb	40.8 (6.9)	41.4 (6.8)
LA limb	40.9 (7.7)	41.2 (8.0)
TDC P2		
MA limb	36.6 (7.7)	36.5 (7.2)
LA limb	32.7 (4.9)	32.6 (5.9)
TDC P3		
MA limb	35.8 (7.3)	35.8 (7.7)
LA limb	32.6 (5.6)	32.9 (6.3)
TDC P4		
MA limb	39.0 (8.3)	38.8 (8.6)
LA limb	32.8 (5.7)	32.6 (6.5)
TDC P5		
MA limb	32.9 (7.7)	33.2 (7.8)
LA limb	29.3 (5.0)	29.5 (5.0)
TDC P6		
MA limb	34.7 (4.0)	35.0 (4.7)
LA limb	33.6 (3.9)	33.3 (3.4)
TDC P7		
MA limb	33.3 (8.4)	32.7 (7.7)
LA limb	28.5 (4.8)	28.7 (6.0)
TDC P8		
MA limb	33.5 (8.4)	33.1 (8.0)
LA limb	30.2 (4.7)	30.2 (5.0)
TDC P9		
MA limb	35.1 (8.6)	35.4 (8.3)
LA limb	31.1 (5.8)	31.4 (5.3)
TDC P10		
MA limb	35.5 (8.6)	36.6 (10.2)
LA limb	30.7 (5.1)	31.3 (5.2)
TDC P11		
MA limb	37.6 (7.3)	38.9 (7.4)
LA limb	36.6 (7.5)	37.9 (7.1)
TDC P12		
MA limb	34.2 (5.6)	34.1 (5.7)
LA limb	31.9 (6.1)	32.2 (6.5)
TDC P13		
MA limb	34.7 (8.6)	34.3 (7.9)
LA limb	30.7 (5.8)	29.8 (4.3)
TDC P14		
MA limb	34.2 (4.6)	34.4 (4.0)
LA limb	32.7 (3.6)	33.0 (3.5)

^a^ LA = less affected; LLL = lower limb lymphedema; MA = more affected; P = measuring point; TDC = tissue dielectric constant.

^b^ n = 41.

The mean values and SDs of measurements for impedance ratio, volume, and TDC from the 2 test occasions in the 42 participants are shown in Table 3. On average, there were 14 days (SD = 2 days) between the 2 test occasions.

**Table 4:** Test–Retest Reliability of Impedance Ratio, Volume, and TDC Measurements for 14 Measuring Points in More and Less Affected Limbs of 42 People With LLL^a^

**Measurement**	**ICC(2,1)**	**95% CI for ICC**	**Mean *d***	**95% CI for Mean *d***	**SEM**	**SEM%**	**SRD**	**SRD%**
Impedance ratio								
MA limb*^b^*	0.90	0.81–0.95	0.034	0.003 to 0.065	0.068	5.2	0.188	14.4
LA limb*^b^*	0.79	0.63–0.88	0.018	−0.014 to 0.049	0.059	5.0	0.164	14.0
Volume, mL								
MA limb	0.99	0.99–1.00	0.52	−56.88 to 57.93	154.9	1.7	429.3	4.6
LA limb	0.99	0.99–1.00	19.64	−27.64 to 66.92	97.0	1.1	268.9	3.1
TDC P1								
MA limb	0.84	0.72–0.91	0.35	−0.87 to 1.56	2.8	6.7	7.7	18.6
LA limb	0.78	0.63–0.88	0.29	−1.32 to 1.90	3.5	8.5	9.7	23.6
TDC P2								
MA limb	0.86	0.76–0.92	−0.09	−1.31 to 1.35	2.9	7.8	7.9	21.6
LA limb	0.71	0.52–0.83	0.12	−1.17 to 1.41	2.6	8.0	7.2	22.1
TDC P3								
MA limb	0.88	0.79–0.93	−0.56	−1.72 to 0.60	2.7	7.5	7.5	20.7
LA limb	0.92	0.86–0.96	0.24	−0.49 to 0.98	1.6	4.9	4.4	13.4
TDC P4								
MA limb	0.96	0.92–0.98	0.06	−0.70 to 0.83	1.6	4.2	4.6	11.7
LA limb	0.87	0.76–0.93	−0.24	−1.24 to 0.77	2.1	6.4	5.8	17.8
TDC P5								
MA limb	0.84	0.72–0.91	0.44	−0.91 to 1.79	3.0	9.1	8.3	25.3
LA limb	0.77	0.61–0.87	0.22	−0.83 to 1.27	2.4	8.2	6.7	22.6
TDC P6								
MA limb	0.71	0.53–0.84	0.05	−0.98 to 1.08	2.2	6.2	6.0	17.1
LA limb	0.68	0.47–0.81	−0.34	−1.26 to 0.58	2.2	6.6	6.1	18.2
TDC P7								
MA limb	0.89	0.81–0.94	−0.45	−1.61 to 0.72	2.7	8.3	7.5	22.9
LA limb	0.90	0.81–0.94	0.16	−0.61 to 0.93	1.5	5.2	4.2	14.5
TDC P8								
MA limb	0.94	0.88–0.97	−0.33	−1.25 to 0.58	2.0	6.1	5.6	16.8
LA limb	0.85	0.73–0.91	0.01	−0.84 to 0.85	1.8	6.0	5.0	16.5
TDC P9								
MA limb	0.95	0.91–0.97	0.07	−0.78 to 0.93	1.9	5.4	5.3	15.0
LA limb	0.89	0.80–0.94	0.31	−0.52 to 1.14	1.9	6.1	5.3	16.9
TDC P10								
MA limb	0.93	0.87–0.96	0.89	−0.20 to 1.98	2.2	6.2	6.2	17.1
LA limb	0.79	0.65–0.88	0.73	−0.30 to 1.75	2.3	7.4	6.4	20.6
TDC P11								
MA limb	0.79	0.64–0.88	1.26	−0.17 to 2.69	3.3	8.5	9.1	23.6
LA limb	0.76	0.59–0.86	1.33	−0.22 to 2.88	3.6	9.7	10.0	26.8
TDC P12								
MA limb	0.87	0.78–0.93	−0.23	−1.11 to 0.65	2.0	5.8	5.5	16.0
LA limb	0.95	0.90–0.97	0.29	−0.35 to 0.93	1.4	4.4	3.9	12.2
TDC P13								
MA limb	0.91	0.85–0.95	−0.46	−1.53 to 0.60	2.6	7.4	7.1	20.5
LA limb	0.86	0.75–0.92	−0.78	−1.59 to 0.03	2.1	7.0	5.8	19.3
TDC P14								
MA limb	0.83	0.70–0.90	0.37	−0.43 to 1.16	1.9	5.5	5.2	15.3
LA limb	0.79	0.64–0.88	0.46	−0.24 to 1.16	1.6	4.9	4.4	13.5

^a^ *d* = difference between test occasion 2 and test occasion 1; LA = less affected; LLL = lower limb lymphedema; MA = more affected; P = measuring point; SEM = standard error of measurement; SEM% = SEM in relative terms; SRD = smallest real difference; SRD% = SRD in relative terms; TDC = tissue dielectric constant.

^b^ n = 41.

### Test–Retest (Intrarater) Reliability

Test–retest reliability data for impedance ratio, volume, and TDC measurements are shown in Table 4. For the impedance ratios, the ICC(2,1) ranged from 0.79 to 0.90 and the 95% CIs were narrow. The mean difference was small in both limbs, and for the more affected limb, a systematic difference in the mean was present, because 0 was not included in the 95% CI. The SEM% was 5.0% for the less affected limb and 5.2% for the more affected limb. The SRD% was 14.0% for the less affected limb and 14.4% for the more affected limbs.

For the volume, the ICC(2,1) values were high (0.99) and the 95% CIs were narrow. The mean difference was small in both limbs, and no systematic differences in the mean were present. The SEM% was 1.1% for the less affected limb and 1.7% for the more affected limb. The SRD% was 3.1% and 4.6% for the less affected and more affected limbs, respectively.

For the TDC, the ICC(2,1) ranged from 0.68 to 0.96. The mean difference was small in all points and no systematic differences in the mean were present. For all points in both limbs, the SEM% ranged from 4.2% to 9.7% and the SRD% ranged from 11.7% to 26.8%.

**Figure 2:**
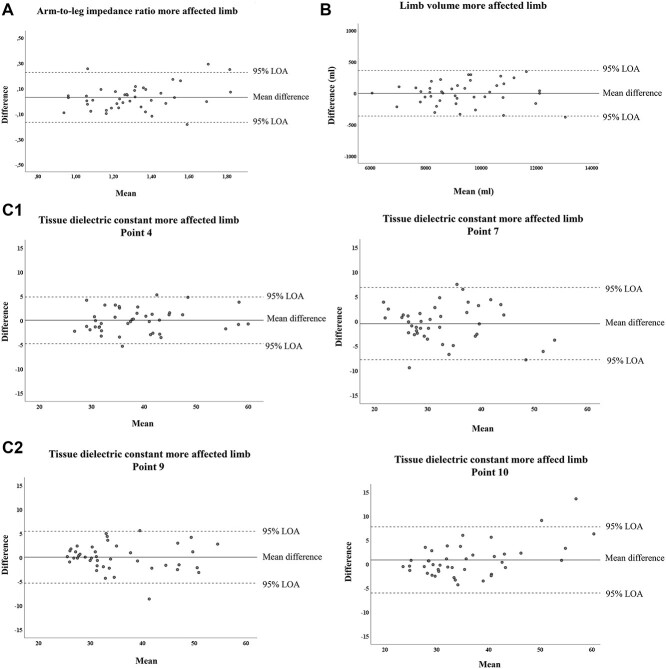
Measurements in the more affected limb in people with lower limb lymphedema: visual illustration of the differences between the test occasions (test 2 − test 1) plotted against the means of the 2 test occasions and the 95% limits of agreement (LOA) for the impedance ratio (A), volume (B), and tissue dielectric constant (TDC) (C, points 4, 7, 9, and 10).

The Bland-Altman graphs for the more affected limb (Fig. 2A–C) show that the differences between the test occasions were small for all 3 measurement methods. For the impedance ratios, generally higher values appeared on the second test occasion (Fig. 2A). For the volume (Fig. 2B) and the TDC at measuring points 4, 7, 9, and 10 (Fig. 2C), no systematic biases or outliers were seen.

## Discussion

To the best of our knowledge this is the first study that has evaluated the test–retest (intrarater) reliability of impedance of ECF, volume, and local tissue water measurements in people with LLL. Overall, we found that the reliability was high and measurement errors were acceptable, both for a group of people and for a single individual.

According to Fleiss,^25^ ICCs above 0.75 represent excellent reliability. In the present study, the reliability of impedance ratio, volume, and TDC measurements were excellent except for P6 [ICC(2,1) = 0.71] in the more affected limb, and for P2 [ICC(2,1) = 0.71] and P6 [ICC(2,1) = 0.68] in the less affected limb. The ICCs for the impedance ratio are similar or slightly better than in other studies of upper limb lymphedema using BIS^29^ (ICC = 0.95) and L-Dex ratio^30^ (ICC = 0.69). The ICCs for the volume are also in line with other studies of volume measurements in upper limbs^29^^,^^31^ (ICCs = 0.97–0.98) and in lower limbs of women and men who were healthy [ICC(2,1) = 0.99].^7^ This indicates that the volumes based on circumference measurements are as reliable in the lower limbs as in the upper limbs. Moreover, the ICCs for the TDC measurements in our study were somewhat higher than in women and men who were healthy [ICC(2,1) = 0.63–0.93 and 0.21–0.89, respectively].^7^ These data indicate that all points are suitable to measure in people with LLL.

Furthermore, the 95% CI for the mean difference was narrow and included 0 for the impedance ratio, volume, and TDC measurements; these results indicated no systematic difference in the mean between the 2 test occasions, except for the impedance ratio in the more affected limb. These data are in line with the volume and TDC measurements in a reliability study of women and men who were healthy.^7^

The measurement errors for a group of people (SEM/SEM%) as well as for a single individual (SRD/SRD%) represent the limits for normal variations of measurement values. Hence, a variation in LLL outside this range indicates a real, clinical change.^27^ In the present study, the SEM values for the impedance ratio were 0.068 for the more affected limb and 0.059 for the less affected limb. These data are consistent with the SEM value (0.06) that was previously presented for the interlimb *R*_0_ ratio in upper limb lymphedema.^29^

For the volume, the SEM values were 97 mL for the less affected limb and 154.9 mL for the more affected limb. These data are in agreement with the SEM values in upper limb lymphedema^29^^,^^31^ (94–78.8 mL) and in the lower limbs of people who were healthy (94.6–120.9 mL).^7^ These results indicate that the volume based on circumference measurements in mild to moderate LLL yields measurement errors as small as those in upper limb lymphedema when the same examiner performs the measurements.

For the TDC, the relative measurement errors were acceptable for all points in both limbs (SEM% = 4.2%–9.7%; SRD% = 11.7%–26.8%). These data are slightly better than those obtained using the same points for the lower limbs of women and men who were healthy (SEM% = 3.9%–14.5%; SRD% = 10.8%–40.1%).^7^ These results indicate that all these points are usable in people with mild to moderate LLL when compression stockings in good condition are used.

When we illustrated the data for the more affected arm-to-leg impedance ratio, volume, and some TDC points in the Bland-Altman graphs, the differences between the test occasions were approximately within the limits of agreement for all 3 measurement methods. The limits of agreement and the SRD are algebraically similar,^14^ but an advantage of calculating SRD% is that it is easier to interpret clinically.

Taken together, the results of the present study indicate that all 3 measurement methods can be used in people with unilateral or bilateral LLL. For the BIS method the curves were checked and considered sufficient. The TDC method is rather time consuming due to both the measurement technique with triplicates at each point and the large number of measuring points chosen to be evaluated in this study. To use this triplicate technique is a matter of clinical consideration, but for LLL this technique has been recommended.^24^ Furthermore, in the present study a large number of measuring points on the calf and thigh were used. The same measuring points have been used in a test–retest reliability in people who were healthy with the intention to cover many different parts of the total limb,^7^ and they were chosen based on our clinical experience of LLL. Previously only 3 measuring points (foot, ankle, and lower limb) have been used when investigating if TDC measurements could differentiate lymphedema from lipedema in swollen legs.^32^ Three measuring points (foot, lateral calf, medial calf) were also used when evaluating the triplicate technique as a standard measurement comparing with duplicate and single measurement.^24^ To use only 3 measuring points would be preferable, but in our study we found higher values (exceeding suggested cutoff values; ie, mean + 3 SDs in a population of people who were healthy)^7^ in 5 measuring points (P4, P7, P9, P10, and P13) in about one-third of the participants, whereas in 3 measuring points (P1, P6, and P11), none of the participants had higher values. These results indicate that higher TDC values could be measured in both the calf and the thigh in people with LLL even though the lymphedema was persistent and new compression garments were used. Which TDC points are clinically relevant for evaluating changes in local tissue fluid over time should be investigated in future studies. However, measuring many anatomical sites enables more individualized management of LLL.

### Clinical Implications

There is a great need of research evaluating measurement properties of instruments for LLL.^33^ Hence, the results from the present study contribute knowledge to an important area. Objective measurements are crucial in LLL management, and measurements from the 3 instruments were therefore analyzed in the studied population. Which measurement methods are to be recommended in LLL management can be a matter of clinical consideration, based on the availability of measurement devices and amount of available measuring time. However, our results indicate that all 3 methods are reliable and can be used to determine effects of an intervention by evaluating changes in these measurements over time in people with mild to moderate LLL. Whether or not these methods also are reliable in severe LLL, with the presence of fibrosis and skin changes that might alter the measurement values, requires further investigation.

### Strengths and Limitations

A strength of the present study was that a highly standardized test protocol was used. A similar protocol was used in a previous test–retest reliability study of volume and TDC in a population of people who were healthy.^7^ To yield a higher probability of stable values, the measurements were conducted in the morning at about the same time, and it was verified that compression stockings no older than 2 months were used. Another strength was that the measurements were conducted by an experienced physical therapist familiar with the different measurement methods, and that all 3 methods evaluated each limb separately. For the volume, it is well known that a weight change could be an aggravating aspect when evaluating LLL over time; using additional measurement methods is therefore warranted. Furthermore, there was on average 14 days between the 2 test occasions, which enabled the natural variation of edema to be taken into account. Even though it could be claimed that the time interval was long for a test–retest reliability study, it is recommended in previous literature.^34^ Also, our results indicate that the time interval was acceptable due to the adequate levels of measurement variation.

A limitation of this study is that interrater reliability was not tested, which should be considered in future studies. Another limitation was the small number of men included; a larger sample size would have enabled data analyses separately for women and men. All participants had mild to moderate LLL, which could be considered a limitation because many people with LLL have larger volumes. The height used for calculation of body mass index was self-stated, and this could have been more objectively assessed. Furthermore, the duration of each test occasion of approximately 75 minutes (10 minutes of rest, 10 minutes for BIS, 15 minutes for circumference measurements, 25 minutes for TDC, and 15 minutes for logistics) was long. To use fewer points when evaluating local tissue water with TDC could be one way of shortening the measuring time.

### Conclusions

Impedance of ECF, volume, and local tissue water can be reliably measured in people with mild to moderate, unilateral or bilateral LLL. The measurement errors were acceptable in all 3 methods (ie, arm-to-leg BIS ratios, volume, and TDC), indicating that real, clinical changes in lymphedema can be measured both in a group of people and in a single individual with LLL.
